# Environmental and individual factors associated with gestational weight gain

**DOI:** 10.1186/s12889-022-12948-w

**Published:** 2022-03-18

**Authors:** Thales Philipe Rodrigues da Silva, Thamara Gabriela Fernandes Viana, Milene Cristine Pessoa, Mariana Santos Felisbino-Mendes, Monique Louise Cassimiro Inácio, Larissa Loures Mendes, Gustavo Velasquez-Melendez, Eunice Francisca Martins, Fernanda Penido Matozinhos

**Affiliations:** 1 Departamento de Enfermagem Materno Infantil E Saúde Pública, Postdoctoral Fellow, Ph.D in Health Sciences, Child and Adolescent Health, Universidade Federal de Minas Gerais. Escola de Enfermagem, Programa de Pós-Graduação Em Enfermagem, Belo Horizonte, MG Brazil; 2 Master in Health and Nursing, Universidade Federal de Minas Gerais. Escola de Enfermagem, Belo Horizonte, MG Brazil; 3 PhD. Professor, Departamento de Nutrição, Universidade Federal de Minas Gerais. Escola de Enfermagem, Belo Horizonte, MG Brazil; 4 PhD. Professor, Departamento de Enfermagem Materno Infantil E Saúde Pública, Universidade Federal de Minas Gerais. Escola de Enfermagem, Belo Horizonte, MG Brazil; 5 Master in Nutrition and Health, Universidade Federal de Ouro Preto. Escola de Nutrição, Ouro Preto, MG Brasil

**Keywords:** Pregnant, Gestational Weight Gain, Built Environment, Food environment, Epidemiology

## Abstract

**Background:**

Environmental factors have an impact on inappropriate food choices and sedentary lifestyle, and both individually and in combination these factors favour improper gestational weight gain (GWG) and consequent maternal and neonatal health problems. The objective of this study was to analyze the environmental and individual factors associated with GWG.

**Methods:**

Data were from “Born in Belo Horizonte: Survey on childbirth and birth”, a hospital-based retrospective cohort of 506 pregnant women with deliveries in public and private maternity hospitals in Belo Horizonte, Minas Gerais. Data were collected via face-to-face interviews from November 2011 to March 2013. The outcome variable of this study was the GWG categorized based on the Institute of Medicine Guidelines. Explanatory environmental variables included the availability and access to food environment and places available for physical activity in the neighborhood. Explanatory individual variables included socioeconomic and demographic, obstetric and childbirth variables. Generalized estimating equations examined the association of environmental and individual factors with insufficient or excessive GWG.

**Results:**

The final sample consisted of 506 mothers. There was 36.4% pregnant women showing excessive GWG and 22.7% showing GWG below the recommended interval. Regarding excessive GWG, there was a positive association with the number of mixed food purchasing establishments close to the place of residence, pre-pregnancy body mass index in the categories of overweight and obesity, arterial hypertension and the private sector as the predominant place for prenatal consultations.

**Conclusion:**

GWG outside of the recommended interval was associated with individual and environmental factors, and most pregnant women had insufficient or excessive gestational weight gain. Such results can complement previously published evidence, important for creating more effective strategies for the prevention of excessive and inadequate GWG and the consequent problems related to it during pregnancy.

## Background

Weight gain is one of the main changes that occurs during pregnancy and gaining excessively or inadequately can have negative implications for maternal and newborn health [1]. Gestational weight gain (GWG) occurs in response to the need for growth and expansion of organs linked to the maintenance of pregnancy and aspects related to the growth and development of the foetus. There is a recommended interval of weight gain for each trimester of pregnancy [2] and this is assessed using the pre-pregnancy body mass index (BMI) of the woman.

Adequate weight gain during pregnancy, according to the recommendations of the Institute of Medicine (IOM) [2], is assessed considering pre-pregnancy BMI and can be influenced by biological, psychological, social and environmental factors [2]. Underweight women should gain 0.5 kg per week in the second and third trimester of pregnancy, achieving total gain of between 12.5 to 18 kg. Among women with adequate BMI, the weekly gain in the second and third trimester should be 0.4 kg with a total gain between 11.5 and 16 kg. For overweight and women with obesity, the gain should be, respectively, 0.3 and 0.2 kg per week in the last two trimesters of pregnancy, the recommended total gain is 7.0 to 11.5 kg for overweight and 5.0 to 9.0 kg for the women with obesity [2].

Insufficient GWG referring to GWG below the recommended interval is related to the development of anemia, maternal protein-energy malnutrition and intrauterine growth restriction [3, 4]. In addition, it can lead to low birth weight and length of the newborn and premature birth [5]. Excessive GWG is associated with gestational diabetes mellitus (GDM), pre-eclampsia and breathing problems for the pregnant woman [3, 4]. There is also an association with macrosomia, cephalopelvic disproportion during labor and increased risk of foetal asphyxia [6, 7].

Guidelines presented by the IOM [2] for the prevention of insufficient GWG or excessive GWG demonstrate the importance of knowing and acting on the risk factors that determine women's nutritional status and pre-pregnancy BMI. It is understood as necessary to assess the individual factors and also the environmental factors that influence weight gain in this period.

The influence of individual factors on GWG, especially on excessive GWG, such as inadequate diet and physical inactivity, is already consolidated in the literature[3, 8, 9]. However, environmental factors have an impact on inappropriate food choices and sedentary lifestyle, and both individually and in combination these factors favor insufficient GWG or excessive GWG and consequent maternal and neonatal health problems [10, 11].

The impact of the characteristics of the community food environment on the health of populations has been analyzed in several studies [11, 12]. Aspects of the built and social environment, including the socioeconomic characteristics of the neighborhood, the availability and accessibility of healthy foods and adequate spaces for physical activity (PA) have been widely associated with the obesity epidemic in the general population in several countries [13–15].

The influence of the built environment (constituted by the physical aspects of the environment that was built or modified by humans) and the social environment (socioeconomic composition and individual and collective living conditions in the neighborhoods), in determining behaviors, such as food choices and physical activity practice has been the focus of studies that have shown the association of the environmental context with the prevalence of overweight and obesity in the general population [16, 17]. Due to the similarity of the outcomes, it is necessary to consider that such behaviors can also impact GWG [10], thus, knowing the repercussions of environmental factors could guide more effective local interventions.

Considering the importance of adequate GWG for the pregnant woman and the foetus and, also, the increase in excess weight in the Brazilian population, including among women of reproductive age. In addition, the scarcity of national studies that consider the influence of the built environment and social status, the objective of this study was to analyze the individual and environmental factors associated with GWG.

## Methods

### Design, study location and period

This is a retrospective cohort, developed with data from the survey “Born in Belo Horizonte: Survey on childbirth and birth”, carried out in 11 maternity hospitals in Belo Horizonte, Minas Gerais(MG), 7 with public care and 4 with private care [18].

The study “Born in Belo Horizonte: Survey on childbirth and birth” adopted the same criteria as the “National Survey: Survey on Childbirth and Birth in Brazil. The research "Born in Brazil: Survey on childbirth and birth" aimed to describe the incidence of caesarean section and examine the consequences on the health of women and new-borns (NB), investigate the relationship between excess caesarean sections, premature delivery and low birth weight and verify the relationship between excess caesarean sections and the use of technical procedures after birth [18].

This study included all women admitted to the maternity hospitals selected at the time of delivery, who had a single pregnancy, adults, residents of Belo Horizonte or Contagem (MG) and who had data on weight and height (necessary for calculating pre-pregnancy BMI). The final sample consisted of 506 mothers.

### Study protocol

The information came from face-to-face interviews, performed by trained nurses, at least 6 h and maximal 24 h after delivery [18], from November 2011 to March 2013. Data from maternal records were also used. More information on the sample design is detailed in other publications [19, 20].

It is noteworthy that the difference in temporality between the years of data collection and the analysis of this proposal will not compromise the results, since it is believed that there was no temporal dissociation in the environmental variables (analyzed in relation to the temporal relationship of the time) and in the buffers design during the study period.

### Outcome variable

The outcome variable of this study was the GWG calculated through the difference between the pre-pregnancy weight and the pre-delivery weight or that registered in the last prenatal care (PN) consultation.

Weight gain was classified based on the IOM's recommendations [2]. According to the pre-pregnancy BMI, pregnant women who had weight gain in the recommended interval were categorized as “adequate GWG”, those who presented weight gain below that recommended for pre-pregnancy were classified as “insufficient GWG” and those who had a gain above the recommended, considering the pre-pregnancy BMI, were categorized as “excessive GWG”.

### Individual explanatory variables

The explanatory variables included in the study were socioeconomic and demographic, obstetric factors and related to childbirth factors (age, ethnicity/race[21], education, marital status, parity, gestational age, pre-pregnancy BMI, smoking in the first 5 months of pregnancy, consumption of alcoholic beverages during pregnancy, number of prenatal consultations, location where most of the prenatal and professional consultations were performed, which attended most of the prenatal consultations). GDM and non-gestational diabetes mellitus, arterial hypertension developed during pregnancy were included as adjustment variables. These variables were chosen as adjustment variables based on previous studies [3, 4].

### Characterization of geographical data

From the home address provided by the participants at the time of data collection, a geographical coordinate (latitude and longitude) was assigned to each study participant. Based on the address and Postal Address Code (CEP) of the location, a geocoded database was developed, obtained through several commercial and government sources to assess the characteristics of the environment built in the buffers. In this way, it was possible to perform the georeferencing of participants and establishments selling the environment built in the Belo Horizonte and Contagem space, as well as the categorization regarding the proximity of their homes to points of sale and food and local stores for the practice of PA. For the process of geocoding the addresses, the GGMAP package was used in R, version 3.4.3.

The union of environmental data, including food outlets and PA practice with individuals, located through their homes and the definition of the buffer, took place through the QGIS program, version 2.18.14.

### Contextual explanatory variables

The geocoding of the addresses of the environmental variables was performed with the GGMAP package in R, version 3.4.3. In this process, the geographical coordinates (latitude and longitude) of the points of sale of food, places where there are PA practices and the individuals' residence were located on a map.

To characterize the exposure of pregnant women to a specific physical and social environment, the concept of neighborhood was used through the delimitation of a buffer with a radius of 500 m. around the residence, used as a centroid. This radius was established based on the fact that the walking time can vary from 10 to 20 min [22].

To characterize the context of the neighborhood, information was obtained on points of sale of food registered according to the National Classification of Economic Activities (CNAE), a standard council that assigns codes of economic activity and defines criteria used by tax authorities in Brazil and the municipalities studied.

The establishments were classified into three categories: 1. Establishments with a predominance of healthy food offerings: where the acquisition of fresh or minimally processed foods represents more than 50% of the total acquisition; 2. Establishments with a predominance of unhealthy food supply: where the purchase of ultra-processed foods represents more than 50% of the total purchase and 3. Mixed food purchasing establishments: where there is a predominance of purchase of culinary preparations or processed foods or where there is no predominance of purchase of fresh / minimally processed foods or ultra-processed foods [23].

The food environment in this study was assessed using the number of healthy, unhealthy and mixed establishments available in the neighborhood environment assigned to each participant. It is representative of the timeframe that participants were pregnant (2011, 2012 and 2013). The establishments around the buffer were classified as mixed establishments: hypermarkets, restaurants, bakeries, dairy retailers, retailers of food products in general, supply of prepared food for home consumption, supermarkets, grocery stores, canteens and mobile food services [23].

The places used for the practice of PA were classified as public and private, such as: squares, gyms, bike paths and other places for this purpose, the data were obtained through government sources.

To characterize the social environment, the nominal income of the census sectors was calculated, which was divided by the number of people residing in the census sectors that made up the buffer, and this value was assigned to each study participant. This variable was categorized into terciles. Neighborhood income and population data were obtained from the demographic database of the Brazilian Institute of Geography and Statistics (IBGE) 2010, Belo Horizonte and Contagem, MG, Brazil.

### Statistical analysis

For the descriptive analysis of the sample, the estimates were presented in proportions (%), with a 95% confidence interval (95% CI). For the quantitative variables, the asymmetry was verified by the Shapiro–Wilk test and the data were presented by means of median and interquartile range (IQ). The correlation between the variables of the buffer food environment was verified using the Spearman correlation test.

To assess the association between independent variables and insufficient GWG or excessive GWG, logistic regression was performed using Generalized Equation Estimation (GEE), and the pregnant women neighborhood was adopted as the aggregation unit, in order not to hurt the assumption of data independence, due to the possibility of sharing the context variables.

For the construction of the logistic regression model with the individual variables, the *p* ≤ 0.20 value obtained in the bivariate analysis was used as a criterion for the inclusion of the variables, in addition to theoretical criteria. Subsequently, the environmental variables elucidated by the literature associated with insufficient GWG or excessive GWG were included. For all analyzes, a significance level of 5% was considered. As an association measure, Odds Ratio (OR) and 95% CI were used. For data analysis, the statistical package Statistical Software for Professional (Stata), version 14.0 was used.

It is noteworthy that due to the high correlation between the variables of the community food environment (healthy, unhealthy and mixed establishments available around the buffer), individual models were built for each of them, thus avoiding collinearity in the models [24]. It is also worth noting that in the models built using Logistic Regression analysis with Generalized Equation Estimation (GEE), the insufficient GWG showed no statistical significance in the multivariate analysis.

The project “Born in Belo Horizonte: Survey on childbirth and birth” was approved by the Research Ethics Committee of the Federal University of Minas Gerais (UFMG), under Opinion CAAE-0246.0.203.000. All puerperal women and directors of each maternity hospital signed the Free and Informed Consent Term, according to the ethical guidelines described in Resolution No. 466, of December 12, 2012, of the National Health Council, which involve research with human beings. All procedures performed in studies involving human participants were in accordance with the ethical standards of the institutional research committee and with the 1964 Helsinki declaration and its later amendments or comparable ethical standards.

## Results

The median age was 29 years (24–33 years), predominantly brown (62.0%) and had completed high school (54.5%). In this research, 76.5% of the puerperal women were married or in a stable relationship. Considering the obstetric characteristics, 53.7% of women were primiparous. Regarding comorbidities, 6.5% of women had GDM and 14.4% had arterial hypertension (Table 1).

**Table 1:** Distribution of sociodemographic and obstetric characteristics of pregnancy

**Sociodemographic and obstetric characteristics**			
	**n (%)**	**CI 95%**	**Median (IQ)**
**Age (years)**			29 (24–33)
**Educational level**			
Elementary School	105 (20.75)	17.42 – 24.51	
High school	276 (54.55)	50.16 – 58.85	
Higher education	125 (24.70)	21.13 – 28.66	
**Ethnicity/Race**^a^			
White	156 (30.83)	26.94 – 35.00	
Black	36(7.11)	5.16 – 9.71	
Brown	304(60.08)	55.73 – 64.27	
Yellow	8(1.58)	0.79 – 3.13	
Indigenous	2(0.40)	0.09 – 1.57	
**Civil status**			
Stable union	387 (76.48)	72.57 – 79.98	
Without companion	119 (23.52)	20.01 – 27.42	
**Smoking in the first 5 months of pregnancy**			
No	473 (93.48)	90.95 – 95.33	
Yes	33 (6.52)	04.66 – 09.04	
**Alcohol consumption during pregnancy**			
No	447 (88.69)	85.60 – 91.18	
Yes	57 (11.31)	08.81 – 14.39	
**Gestational diabetes**			
No	472 (93.47)	90.94 – 95.32	
Yes	33 (6.53)	04.68—09.06	
**Non-gestational diabetes**			
No	228 (96.61)	93.33 – 98.30	
Yes	8 (3.39)	01.69 – 06.66	
**Pre-eclampsia**			
No	431 (85.18)	81.79 – 88.02	
Yes	73 (14.43)	11.61 – 17.78	
**Parity**			
Primiparous	272 (53.75)	49.38 – 58.07	
Multiparous	234 (46.25)	41.92 – 50.62	
**Gestational age (weeks)**			39 (38–40)
**Number of PN consultations**			
≥ 6	414 (92.20)	89.32 – 94.35	
< 6	35 (7.80)	05.64 – 10.67	
**Professional who attended predominantly PN consultations**			
Nurse	48 (9.49)	07.21 – 12.37	
Physician	458 (90.51)	87.62 – 92.78	
**Place where PN consultations were predominantly carried out**			
Public	269 (55.69)	51.21 – 60.08	
Private	214 (44.31)	39.91 – 48.78	

*CI95%* 95% confidence interval, *IQ* interquartile range, *PN* prenatal care; ^a^ This classification was made with reference to: Ethnicity/race was categorized using the definition used by the ethnic and racial characteristics of the population: a study of the categories of color or racial classification: 2008 from Instituto Brasileiro de Geografia e Estatística (IBGE) [21]

Regarding pre-pregnancy BMI, 23.5% of women had obesity and 11.1% were overweight. A total of 59.1% of pregnant women in the sample had insufficient GWG or excessive GWG, with 36.4% of them showing excessive GWG and 22.7% showing GWG below the IOM's recommendations.

Considering the environmental variables (public and private places available for the practice of PA, number of establishments with healthy food, number of establishments providing unhealthy food, number of mixed food purchasing establishments and the average income of the buffer with a radius of 500 m from the woman's residence), the average of establishments according to the density of establishments per tercile within the 500-m buffer are shown in Table 2. The average income per buffer was R $ 890.42 (95% CI 844.46—936.38), varying between 266.86 and 4688.27 reais.

**Table 2:** Average number of establishments per second tertile of the Buffer with a radius of 500 m, the centroid being the residence of the pregnant woman

	***Mean (SD)***	***Minimum and maximum range***
***Buffer from healthy establishments***		
* 1 Tercile*	2.29 (1.37)	0 – 4
* 2 Tercile*	5.81 (0.80)	5 – 7
* 3 Tercile*	12.08 (9.83)	8 – 121
***Buffer from unhealthy establishments***		
* 1 Tercile*	5.13 (2.59)	0 – 9
* 2 Tercile*	12.76 (1.88)	10 – 16
* 3 Tercile*	30.37 (36.29)	17—393
***Buffer of mixed food establishments***		
* 1 Tercile*	3.46 (1.96)	0 – 6
* 2 Tercile*	9.38 (1.67)	7 – 12
* 3 Tercile*	24.50 (23.11)	13—174
***Buffer locations available for PA practice***		
* 1 Tercile*	0.52 (0.50)	0 – 1
* 2 Tercile*	2.00 (0.00)	2 – 2
* 3 Tercile*	3.71 (0.93)	3 – 7

*SD* standard deviation, *PA* physical activity

Source: prepared for the purposes of this study

Bivariate analyses revealed that pre-pregnancy obesity (*p* = 0.001) and the absence of gestational diabetes (*p* = 0.018) were associated with insufficient GWG. Factors associated with excessive GWG were pre-pregnancy overweight (*p* < 0.001), obesity (*p* < 0.001), arterial hypertension (*p* < 0.001), number of prenatal consultations less than 6 (*p* = 0.039) and the private sector, as the place where most PN consultations were performed (*p* = 0.007). Regarding environmental characteristics, the number of places available for the practice of PA in the second tercile of the distribution was associated with excessive gestational weight gain (Table 3).

**Table 3:** Bivariate analysis for insufficient and excessive gestational weight gain according to sociodemographic, obstetric and environmental factors. Belo Horizonte and Contagem, Minas Gerais

**Sociodemographic, obstetric and environmental factors**						
	**Insufficient weight gain n (%)**	**OR(CI95%)**	***p*- value**	**Excessive weight gain n (%)**	**OR(CI95%)**	***p*- value**
**Age (years)**	115 (22.73)	1.00 (0.96–1.03)	0.995	184 (36.36)	0.98 (0.95–1.01)	0.278
**Educational level**						
Elementary School	24 (22.86)	1		40 (38.10)	1	
High school	61 (22.10)	0.98 (0.54–1.78)	0.965	109 (39.49)	1.14 (0.68 – 1.90)	0.610
Higher education	30 (24.00)	0.79 (0.40 – 1.57)	0.517	35 (28.00)	0.65 (0.36 – 1.19)	0.171
**Ethnicity/Race**^a^						
White	34 (21.79)	1		51 (32.69)	1	
Black	11 (30.56)	1.90(0.76 – 4.73)	0.163	13 (36.11)	1.41 (0.59 – 3.33)	0.429
Brown	66(21.71)	1.06 (0.63 – 1.76)	0.814	117(38.49)	1.31(0.85 – 2.04)	0.215
Yellow	2(25.00)	1.10 (0.18 – 7.46)	0.872	3(37.50)	1.59(0.31 – 8.13)	0.575
Indigenous	2(100)	-		-	-	
**Civil status**						
Stable union	87 (22.48)	1		134 (34.63)	1	
Without companion	28 (23.53)	1.35 (0.78 – 2.34)	0.271	50 (42.02)	1.33 (0.84 – 2.12)	0.215
**Pre-pregnancy body mass index (BMI)**						
Eutrophic	69 (22.33)	1		80 (25.89)	1	
Underweight	9 (40.91)	2.14 (0.83 – 5.48)	0.113	3 (13.64)	0.55 (0.14 – 2.14)	0.392
Overweight	21 (17.65)	1.74 (0.92 – 3.28)	0.087	70 (58.82)	**5.10 (3.08 – 8.44)**	** < 0.001**
Obesity	16 (28.57)	**4.13 (1.74 – 9.79)**	**0.001**	31 (55.36)	**6.65 (3.14 – 14.08)**	** < 0.001**
**Smoking in the first 5 months of pregnancy**						
No	108 (22.83)	1		166 (35.10)	1	
Yes	7 (21.21)	1.58 (0.55 – 4.47)	0.387	18 (54.55)	2.29 (0.99 – 5.27)	0.051
**Alcohol consumption during pregnancy**						
No	104 (23.27)	1		158 (35.35)	1	
Yes	11 (19.30)	0.91 (0.42 – 1.96)	0.817	25 (43.86)	1.36 (0.73 – 2.52)	0.318
**Gestational diabetes**						
No	104 (22.03)	1		171 (36.23)	1	
Yes	11 (33.33)	2.15 (0.88 – 5.25)	0.091	12 (36.36)	1.30 (0.55 – 3.06)	0.539
**Non gestational diabetes**						
No	44 (19.30)	1		89 (39.04)	1	
Yes	6 (75.00)	13,16 (1.56 – 111.15)	**0.018**	1 (12.50)	1,25 (0.07 – 19.90)	0.871
**Parity**						
Primiparous	47 (23.50)	1		72 (36.00)	1	
Multiparous	49 (21.78)	0.97 (0.58 – 1.60)	0.912	90 (40.00)	1.45 (0.96 – 2.21)	0.076
**Gestational age (weeks)**	115 (22.73)	0.97 (0.85 -1.10)	0.649	184 (36.36)	0,97 (0.88 – 1.06)	0.579
**Number of PN consultations**						
Greater than or equal to 6	95 (22.95)	1		142 (34.30)	1	
Less than 6	8 (22.86)	1.62 (0.60 – 4.35)	0.333	18 (51.43)	**2.36 (1.04 – 5.34)**	**0.039**
**Professional who attended most PN consultations**						
Nurse	15 (31.25)	1		15 (31.25)	1	
Physician	100 (21.83)	0.62 (0.30 – 1.30)	0.221	169 (36.90)	1.14 (0.56 – 2.32)	0.705
**Place where most PN consultations were carried out**						
Public	63 (23.42)	1		112 (41.64)	1	
Private	48 (22.43)	0.67 (0.41 – 1.08)	0.104	65 (30.37)	**0.57 (0.37 – 0.86)**	**0.007**
**Healthy food purchasing establishments**						
1 tercile	52 (24.19)	1		74 (34.42)	1	
2 tercile	27 (19.01)	0.70 (0.40 – 1.25)	0.238	54 (38.03)	1.03 (0.64 – 1.65)	0.900
3 tercile	36 (24.16)	1.06 (0.61 – 1.83)	0.834	56 (37.58)	1.08 (0.68 – 1.71)	0.736
**Unhealthy food purchasing establishments**						
1 tercile	41 (23.03)	1		56 (31.46)	1	
2 tercile	33 (20.63)	1.01 (0.57 – 1.77)	0.961	62 (38.75)	1.30 (0.81 – 2.10)	0.267
3 tercile	41 (21.40)	1.27 (0.72 – 2.23)	0.393	66 (39.29)	1.45 (0.91 – 2.30)	0.109
**Mixed establishments near the place of residence**						
1 tercile	42 (23.08)	1		57 (31.32)	1	
2 tercile	35 (21.47)	1.05 (0.60 – 1.84)	0.842	63 (38.65)	1.34 (0.83 – 2.16)	0.226
3 tercile	38 (23.60)	1.20 (0.68 – 2.13)	0.514	64 (39.75)	1.47 (0.92 – 2.34)	0.103
**Available places for physical activity**						
1 tercile	67 (23.51)	1		95 (33.33)	1	
2 tercile	21 (20.39)	1.02 (0.54 – 1.93)	0.935	46 (44.66)	1.79 (1.07 – 2.97)	**0.024**
3 tercile	27 (22.88)	1.02 (0.58 – 1.79)	0.935	43 (36.44)	1.10 (0.69 – 1.74)	0.670
**Average income per Buffer**						
1 tercile	31 (20.39)	1		53 (34.87)	1	
2 tercile	39 (22.67)	1.35 (0.75 – 2.44)	0.311	70 (40.70)	1.45 (0.90 – 2.34)	**0.122**
3 tercile	45 (25.00)	1.23 (0.68 – 2.20)	0.485	61 (33.89)	1.02 (0.65 – 1.61)	0.911

*P*-value for Logistic Regression Models with Generalized Equation Estimation / 95% CI = 95% Confidence interval/Bold values =  < 0.2/ *OR* Odds Ratio, *PN* Prenatal; ^a^ This classification was made with reference to: Ethnicity/race was categorized using the definition used by the ethnic and racial characteristics of the population: a study of the categories of color or racial classification: 2008 from Instituto Brasileiro de Geografia e Estatística (IBGE) [21]

Due to the high correlation between the variables of the community food environment they were included in separate models.

In the models constructed using the Logistic Regression analysis with Generalized Equation Estimation (GEE) (Table 4), insufficient GWG did not show statistical significance in the multivariable analysis.

**Table 4:** Logistic Regression Models with Generalized Equation Estimation (GEE) for excessive gestational weight gain according to sociodemographic, obstetric and environmental factors. Belo Horizonte e Contagem, Minas Gerais

**Sociodemographic, obstetric and environmental factors**									
**Excessive gestational weight gain (*n* = 115)**									
	**Model 1**			**Model 2**			**Model 3**		
	**OR**	**CI95%**	***p*- Value**	**OR**	**CI95%**	***p*- Value**	**OR**	**CI95%**	***p*- Value**
**Age**	0.96	0.92 – 1.01	0.144	0.96	0.92 -1.01	0.128	0.96	0.91 – 1.00	0.092
**Educational level**									
Elementary School	1	1		1	1		1	1	
High school	1.30	0.67 – 2.55	0.432	1.35	0.69 – 2.62	0.376	1.39	0.71 – 2.71	0.334
Higher education	0.78	0.34 – 1.83	0.583	0.76	0.33 – 1.78	0.541	0.79	0.34 – 1.85	0.593
**Smoking in the first 5 months of pregnancy**									
No	1	1		1	1		1	1	
Yes	2.73	0.98 – 7.58	0.054	2.79	**1.02 -7.64**	**0.045**	2.73	0.98 – 7.54	0.052
**Pre-gestational body mass index (BMI)**									
Eutrophic	1	1		1	1		1		
Underweight	0.66	0.16 – 2.66	0.562	0.64	0.15 – 2.57	0.531	0.60	0.14 – 2.53	0.493
Overweight	5.74	**3.13 – 10.51**	** < 0.001**	5.76	**3.15 – 10.53**	** < 0.001**	6.24	**3.37 – 11.54**	** < 0.001**
Obesity	8.39	**3.33 – 21.09**	** < 0.001**	8	**3.22 – 19.90**	** < 0.001**	8.37	**3.32 – 21.06**	** < 0.001**
**Number of prenatal consultations**	1.01	0.92 – 1.11	0.739	1.01	0.93 – 1.11	0.673	1.02	0.93 – 1.12	0.614
**Gestational age**	1.01	0.92 – 1.11	0.815	0.98	0.86 – 1.12	0.852	0.99	0.86 – 1.13	0.892
**Professional**									
Nurse	1	1		1	1		1	1	
Physician	1.22	0.49 – 3.01	0.656	1.12	0.45 – 2.77	0.797	1.11	0.45 – 2.76	0.811
**Unhealthy food purchasing establishments**									
1 tercile	1	1							
2 tercile	1.45	0.80 – 2.63	0.217						
3 tercile	1.91	**1.00 – 3.64**	**0.047**						
**Healthy food purchasing establishments**									
1 tercile				1	1				
2 tercile				1.24	0.68 – 2.25	0.481			
3 tercile				1.18	0.65 – 2.11	0.573			
**Mixed food purchasing establishments near the place of residence**									
1 tercile							1	1	
2 tercile							1.36	0.72 – 2.55	0.337
3 tercile							2.71	**1.35 – 5.43**	**0.005**
**Average income per Buffer**									
1 tercile	1	1		1	1		1	1	
2 tercile	1.45	0.79 – 2.68	0.227	1.58	0.86 – 2.90	0.133	1.28	0.68 – 2.41	0.443
3 tercile	1.04	0.53 – 2.05	0.902	1.33	0.71 – 2.48	0.363	0.78	0.38 – 1.61	0.515

^*^P-value for Logistic Regression Models with Generalized Equation Estimation; /95% CI = 95% Confidence interval/*OR* Odds Ratio/*BMI* body mass index, *PN* prenatal care. /*Ref* reference category/Values in bold =  ≤ 0.05. Non-gestational diabetes and arterial hypertension were considered as adjustment variables of the model

With regard to excessive GWG, an association was observed with the largest number of mixed food purchasing establishments near the place of residence (*p* = 0.005). That is, women who lived in regions with the highest number of mixed food establishments had more than twice the chance of having a higher GWG when compared to those who lived in less densely populated areas. Also, pre-pregnancy BMI in the categories of overweight and obesity (*p* < 0.001), arterial hypertension (*p* = 0.007) and the location of PN consultations in the private sector (*p* = 0.018) in model 1, were associated with excessive GWG.

## Discussion

This study found a positive association between GWG and the number of mixed food purchasing establishments close to the place of residence, pre-pregnancy BMI in the categories of overweight and obesity, arterial hypertension and the private sector as the predominant place for prenatal consultations.

The results of this study showed a high percentage of insufficient GWG[2]. It was greater among women with obesity in pre-pregnancy. Previous study presented similar findings [25], which demonstrated the need for interventions that contribute to adequate GWG, since the risks of insufficient GWG or excessive GWG, increases the chance of negative maternal and neonatal outcomes [3, 4]. PN consultations can be an opportunity for this intervention and counselling since they occur throughout the pregnancy, allowing multiple approaches to adequate GWG.

It was observed that women with overweight and pre-gestational obesity were at risk of having excessive GWG. Obesity and overweight pre-pregnancy, as evidenced by BMI, show an accumulation of risk factors, and there may be a synergistic effect of these negative factors to excessive GWG and, consequently, on reproductive outcomes [3, 4, 7]. Previous studies have already shown that obesity prior to pregnancy can lead to an increased risk of maternal and neonatal complications, such as GDM, high blood pressure developed during pregnancy, pre-eclampsia, eclampsia, thromboembolic phenomena, urinary infections, preterm birth, dystocic births that increase the incidence of caesarean sections, foetal malformations, foetal macrosomia, foetal death, massive postpartum haemorrhage, puerperal infection, maternal death, among others [25–27].

With regard to environmental aspects, the results of this study showed that women who lived in areas with the largest number of establishments selling food products considered unhealthy have almost twice the chance of having a higher GWG when compared to women, who lived in less densely populated areas. Regions with a high density of establishments that sell foods that are considered unhealthy, especially in urban regions, contribute to the consumption of ultra-processed products due to the availability and access to stores selling these foods, which contributes to a positive energy balance and, consequently, to a greater weight gain in the general population [28, 29]. In addition, pregnant women who live in neighbourhoods with a high prevalence of fast food restaurants has an increased risk of developing GDM [30]. Also, those who live in food deserts greater risk for developing preeclampsia and preterm labor [31]. On the other hand, another study showed that built environments with more healthful food availability were associated with a lower odd of excessive or insufficient GWG [32]

Despite the scarcity of studies evaluating the environmental context and excessive GWG, there is scientific evidence that links the prevalence of women with excessive GWG with the area of residence in urban regions [1, 4, 27, 33].

In this study, women who lived in regions with the highest number of mixed food purchasing establishments have more than twice the chance of having a higher GWG when compared to those who lived in less densely populated areas. The influence of the mixed establishment on excessive GWG is understood, since the food choices of these women are influenced by other factors (in addition to only a healthy lifestyle), with an important association between environment and access, for example. In addition, the characteristic of mixed food purchasing establishments has not yet been well explored by studies. As they are establishments where there is no predominance of healthy or unhealthy foods, better understanding the influence of this type of establishment on food choices is essential.

Although other studies have verified the food environment in urban areas as potentially favoring a sedentary lifestyle [10, 34, 35]. In the social context, the average income of the buffers was also not associated with insufficient GWG or excessive GWG, unlike studies that showed that social and economic disparities around pregnant women, such as the poverty rate in the neighbourhood of residence, can influence the excessive gain during pregnancy [4, 34].

Finally, some limitations in this study must be recognized. Interviews were conducted retrospectively, and thus may be subject to recall bias. The IOM recommendations has its own limitation since they do not take into account fat distribution and other important weight aspects [36, 37]. Another limitation of this study is that the address evaluated was the last one reported by the parturient, and it was not possible to consider changes of address during pregnancy that could modify the parturient's environment. In addition, in relation to some variables, no statistical significance was found in this research—probably due to the small sample size. Added to this aspect, the loss of some data, intrinsic to the fact that data collection was also carried out in medical records. On the other hand, sensitivity analyses were carried out, noting that this aspect would not be significantly affecting the estimates, at least regarding the general conclusions.

Despite these limitations, this work advances in the perspective of analysing data that have not yet been fully explored on women's health. Based on our novel findings focusing in on environmental factors instead of just individual influences on diet and physical activity, future clinical trials in pregnant women at risk of excessive GWG should consider aspects of women’s environment such as the built food environment and offer strategies to improve health behaviours accordingly.

## Conclusion

The findings of this study showed that the environmental context, especially related to the types of trade in food sales, and the pre-pregnancy BMI of pregnant women, can influence excessive GWG and, consequently, the negative outcomes for maternal and child health.

Such results can complement previously published evidence, important for creating more effective strategies for the prevention of excessive GWG and the consequent problems related to it during pregnancy. The findings of this research demonstrate the need for micro and macroenvironment factors analysis in further studies considering their close associations with GWG and their potential influence on maternal and neonatal health. Microenvironment factors generally refer to the space where pregnant women live and go on a daily basis (e.g., home, workplaces, schools, and supermarkets) while macroenvironment factors are the factors influencing pregnant women´s life (e.g., technologies, media, transport system, food industry and urban development) [29].

## Data Availability

The datasets used and/or analysed during the current study will be available from the corresponding author on reasonable request.
